# Marketing in the anthropocene: A future agenda for research and practice

**DOI:** 10.1007/s13162-025-00300-5

**Published:** 2025-03-20

**Authors:** Nancy M. P. Bocken, Laura Niessen, Maike Gossen, Ankita Das, Maria Zielińska

**Affiliations:** 1 Maastricht Sustainability Institute, School of Business and Economics, Maastricht University, Tapijn 11 Building D, P.O. Box 616, 6200 MD Maastricht, The Netherlands; 2 The International Institute for Industrial Environmental Economics (IIIEE), Lund University P.O, Box 196, 22100 Lund, Sweden; 3 Department of Economic Education and Sustainable Consumption, Technical University Berlin, Marchstraße 23, 10587 Berlin, Germany

**Keywords:** Marketing, Strong sustainability, Sufficiency, Climate change, Regeneration, Anthropocene, Research agenda

## Abstract

Marketing is an important function and practice in everyday business. It involves getting potential customers interested in a product or service through value-oriented arguments. In this way, marketing plays a pivotal role in driving the consumption of goods and services. Given the increasing consumption of goods and services, decreasing product lifetimes, and increasing levels of waste in all product categories, it is evident that the practice and theory of marketing needs a radical rethink in light of pressing resource and climate issues. The impact of unsustainable production and consumption patterns has led to this era being referred to as the Anthropocene, in which humans have become the dominant influence on the climate and the natural environment. There is an urgent need to take a new direction to adapt marketing theory and practice to these pressing global needs. In this study, we investigate the following questions: *What role should marketing play in the era of the Anthropocene? What concepts, outcomes, tools and theories does marketing offer to support a transition towards Marketing in the Anthropocene?* We conduct a scoping literature review based on different research directions and propose a conceptualization for “Marketing in the Anthropocene” as an inspirational, forward-looking concept, tool and practice for marketers and marketing researchers. We highlight relevant marketing tools and theories and provide guiding questions for future research and practice.

## Introduction

“People are persuaded to spend money we don't have, on things we don't need, to create impressions that won't last, on people we don't care about.”

― Tim Jackson (2009)

Since the world has already crossed six planetary boundaries and is also facing extensive environmental degradation and increasing extreme weather events (Richardson et al., 2023; Rockström et al., 2023), this era is dubbed the “Anthropocene” where humans are shaping earth’s geophysical cycles and the relations with the living world through consumption and production processes (Steffen et al., 2011).^1^ Humans have been creating a world with large uncertainties and increasing droughts, floods and other climate change-related effects (Lee et al., 2023) and a world where there is more plastic in the oceans than fish (World Economic Forum, Ellen MacArthur Foundation & McKinsey & Company, 2016). Certainly, the last decades have seen significant progress in terms of poverty reduction, health care and education and it should be recognised that there is some form of “good Anthropocene” to be preserved (Rosling et al., 2019). Yet, while some argue that the Anthropocene is mainly good and certainly not a “time of crisis” (see Ellis, 2011), there is ample evidence that shows that the great acceleration of consumption and production of goods has led to devastating effects on living habitats and the climate (McPhearson et al., 2021). Hence, the “bad Anthropocene” and human-made impact need to be mitigated (Christian, 2018; Hamilton,  2016; Steffen et al., 2015). Radical change is needed in knowledge, technology, institutions, and new ways of doing business, as well as personal behaviour and meanings (McPhearson et al., 2021). This radical change therefore applies to the dominant ways in which we produce and consume products and services where we both need to question *how* as well as *how much* we consume, in particular in affluent countries. How and how much we consume needs to be tackled to mitigate human impact in the “bad Anthropocene.” In terms of marketing, the business function which is at the core of driving unsustainable consumption patterns (Pereira Heath & Chatzidakis, 2012), this could positively translate into a “[m]oderation of marketing and retail activities, choice editing, quality durable products, repair and reuse" (Bocken & Short, 2021, p. 3). New regenerative ways of producing and consuming need to be introduced to further build out the “good Anthropocene” with decent ways of living for all. In terms of marketing, this will involve the creation of positive impacts through products or services introduced in the market (Konietzko et al., 2023).

The radical changes required for transforming pathways towards a “good Anthropocene” indicate a core role for marketing as a business function. As an everyday practice in business and the process of getting stakeholders or potential customers interested in the product or service through value-based arguments (Kotler, 2011), the marketing concept indicates meeting the needs and requirements of consumers and creating added value for society at large. However, in its most dominantly used form in practice, mainstream marketing tools and tactics often drive excessive consumption (Achrol & Kotler, 2012; Assadourian, 2010; McDonagh & Prothero, 2014; Stoeckl & Luedicke, 2015; Varey, 2010) and therefore unsustainable production systems of goods and services, with associated waste and emissions in most sectors (Bocken & Short, 2021). Critics complain that marketing practice is inextricably tied to the capitalist system (Ardley & May, 2020). As a result, marketing pushes products that are low quality, do not perform well or are sometimes even harmful and unsafe. Consumers are led to buy items in buy-one-get-one-free offers and to buy at an ever-increasing pace adding to the environmental footprint, fueled by marketing campaigns enticing them to buy more than they need (Bocken & Short, 2021; Peattie & Peattie, 2009).

As a consequence, marketing is typically the business function most often charged with ethical abuse (Smith et al., 2012). Marketing issues such as dishonest advertising, cheating customers and overselling, have been noted as major ethical marketing-related problems (Chonko & Hunt, 1985). Marketing ethics has evolved as a subfield of business ethics to study “how moral standards are applied to marketing decisions, behaviors, and institutions” (Laczniak, 2012, p. 208). The assumption is that it is morally wrong for society to engage in activities that pollute and destroy the economic, natural and social environment. This has led to evolving ethical norms and values, as stipulated by the American Marketing Association (AMA), which recognizes that “the marketing community not only serves organisations but also acts as a steward of society in creating and facilitating the transactions and experiences that drive the greater economy” (American Marketing Association, 2024). Hence, one would think that marketing, with increasing knowledge of environmental and societal issues, would have improved its challenging role. Yet, Murphy et al. (2017) note several recent marketing scandals, for instance the exploitation of megatrends such as environmental sustainability and health through false marketing messages (“greenwashing”) (Murphy et al., 2017). A review by the UK Competition and Markets Authority and the Netherlands Authority for Consumers and Markets found that 40% of sustainability claims by firms can be misleading, for instance by using own brand eco labels or hiding or omitting information about the product’s pollution levels (UK Government Competition and Markets Authority, 2021). For example, a legal complaint was issued to the European Commission in 2023 demanding a review of Coca-Cola, Nestle and Danone for claiming that their plastic bottles were “100% recycled,” a state which is not technologically possible (Legget & Edser, 2023). In the fashion industry, using private sustainability labels with false information and ambiguous terms that cause confusion among consumers and lead to misinterpretation of environmental claims is a common practice (Gossen et al., 2022). Several unethical marketing actions are arguably legal, so it is important to understand that ethics always has personal, organisational, and societal implications (Murphy et al., 2017). Hence, this opens up a discussion of a new normative perspective on marketing that better copes with increasing environmental and societal issues.

As a response, sustainable marketing aims “to achieve economic viability, ecological health, social equity, widespread moral practices, and technological advancement and adoption” (Lim, 2016, p. 243). In this holistic definition, sustainable marketing goes beyond the triple bottom line approach by incorporating the ethical and technological dimension (Lim, 2016), and can be applied to a variety of stakeholders such as individuals, companies, governments, non-governmental organisations and local communities. By responding to basic needs, it enhances the quality of life and well-being of current and future generations (Lunde, 2018). In the past, scholars have pointed out that most sustainable marketing approaches lack relevance, conceptual framing and theoretical clarity (e.g., Gordon et al., 2011; Kilbourne, 2004; Lunde, 2018), and focus on the weak sustainability concept leading to unreliable approaches and even giving rise to terms such as greenwashing, which contributes to the general mistrust of marketing. This is mirrored by marketing research focusing on micro-level and insulated issues rather than contextualised systemic issues and outcomes (Press, 2021). As a result, a dominant part of literature still focuses on marketing for “business as usual,” not questioning how and how much we consume, which would be urgently needed in light of the Anthropocene.

Behind this backdrop, we argue for a fundamental revamp of marketing practice. Marketing needs to break new ground for conceptually sound solutions to better fit the new reality of the Anthropocene, where business as usual does not serve society and the planet anymore. This echoes the work, for example, by Press (2021) who calls for a strong sustainability research program. From a strong sustainability perspective, humans and the economic system are reliant on the natural environment and man-made capital cannot replace lost natural resources. Therefore, strong sustainability in marketing research would include rethinking business and marketing (theory) to fit a “dramatically resource constrained, high risk environment” (Press, 2021, p. 104). By using the tools, tactics and theories of marketing in a way that serves strong sustainability, the marketing practice is proposed to undergo a fundamental change in the age of the Anthropocene. We investigate the following research questions:

*What role should marketing for strong sustainability play in the era of the Anthropocene? What concepts, outcomes, tools and theories does marketing offer to support a transition towards Marketing in the Anthropocene?*

We attempt to answer these research questions by reviewing and analyzing previous research on marketing strategies and practices contributing to strong sustainability. Therefore, the keywords chosen for the scoping literature review tend to come from an environmental sustainability perspective. This study aims to make the following contributions. First, it contributes a novel forward-looking concept of Marketing in the Anthropocene building on the strong sustainability approach. Second, it presents a future agenda for marketing research. Finally, it presents a roadmap for business practice and policy.

The remainder of the paper is structured as follows. First, the background of our study is provided and the current state of research on sustainability marketing and the changes that a strong sustainability approach requires are discussed. Second, the scoping literature review method is introduced. After this, the results are presented, organised starting with a general overview of the articles analysed, followed by the marketing outcomes, tools and theories. Finally, we end with a discussion on the “eras of marketing,” a novel framework for Marketing in the Anthropocene, and a discussion on next steps for key actors, followed by short conclusions, including suggestions for future research and practice.

## Background

### Sustainability in marketing

The marketing discipline recognised environmental concern already in the 1970s (Peattie, 2001). It has evolved from a narrower focus of ecological marketing (reducing the dependency on products causing environmental harm), to environmental marketing and green consumerism, and sustainable marketing that embeds environmental cost of production and consumption into marketing practice (Peattie, 2001). In the early 2010s, leading marketing journals began to create special issues on sustainability marketing, such as the Journal of the Academy of Marketing Science's special issue on sustainability in 2011,^2^ which led to a significant increase in research on sustainable marketing.

In a recent systematic literature review published in this journal, Lunde (2018) examined scientific articles on the topic of sustainability published in the most important marketing journals over a period of 20 years up to 2016. He found that marketing research on sustainability has been fragmented in the past, illustrated by the lack of a standardised definition of sustainable marketing. By sorting and summarising the existing research, he outlined five sustainability principles that form the GREEN Framework of Sustainable Marketing. These principles are: Globalised marketplace of value exchange; responsible environmental behaviour for current and future generations; equitable sustainable business practices; ethical sustainable consumption; necessary Quality of Life (QOL) and well-being (Lunde, 2018). Although the framework addresses the need to develop a view of sustainable marketing as a macro and holistic concept, the need for a more normative perspective on marketing that better addresses increasing environmental and societal challenges is largely overlooked in the literature. In line with this, Lloveras et al. (2022) state that sustainable marketing allowed a continued focus on affluent lifestyles without considering more radical forms such as less consumption. Hence, most marketing practices operate within the “weak” sustainability paradigm, which assumes that natural and manufactured capital can replace each other and that small changes in the system should suffice. In businesses, weak sustainability is echoed in the rhetoric of the business case for sustainability, which suggests that sustainability efforts will enhance a firm’s bottom line by fostering reputation and customer loyalty. Press (2021) argued that a lot of sustainability work in marketing is focused on “the trees […] rather than the forest” (p. 96) and demanded a strong sustainability approach to marketing. She clarified that a strong sustainability approach in marketing research “addresses issues and tensions associated with strong sustainability” (p. 98) and should view the biosphere as the base of society and the economy. A strong sustainability approach thus recognises the limits to planetary resources and the non-substitutability of natural capital through money (Dietz & Neumayer, 2007).

The call for strong sustainability is accompanied by various counter movements in marketing research and practice. Some of the underlying ideas of strong sustainability, such as moderating and reducing consumption through marketing, are not exactly new in marketing research and practice. Fisk (1973) was one of the first marketing scholars to discuss the need to limit consumption, followed by several studies that investigated social marketing for consumption reduction (e.g., Lahtinen et al., 2020; Peattie & Peattie, 2009), green demarketing (e.g., Armstrong Soule & Reich, 2015; Reich & Armstrong Soule, 2016; Lawrence and Mekoth, 2023) and sufficiency-promoting marketing (e.g., Gossen et al., 2019), as well as regenerative business practice, which is about doing more net good rather than less bad (Konietzko et al., 2023; Polman & Winston, 2021).

Our study builds on this pioneering work and follows the attempts and calls of previous research for a more holistic and comprehensive understanding of sustainability in the marketing literature (Lunde, 2018) and for transformative and reformative approaches (Kemper & Ballantine, 2019), and pays more attention to the far-reaching macro relationships between marketing and the natural environment (McDonagh & Prothero, 2014).

### Theories and tools of sustainability marketing

Innovations in marketing concepts are typically associated with the renewal of marketing tools and theories.

The earlier conceptualisation of marketing in a business context goes back to Jerome McCarthy in 1960 who defined the marketing mix as the tool which the company seeks to combine to satisfy specific target groups, including product, price, place, and promotion (4Ps) (McCarthy, 1960). The 4Ps have been widely used, but also criticised. Yet, some research with a sustainability focus still applied the 4Ps such as in the case of social marketing (e.g., Lahtinen et al., 2020; Varey, 2002; Sheth et al., 2011; Gordon et al., 2011; McDonagh & Prothero, 2014) and marketing for reduced consumption (Gossen & Kropfeld, 2022; Peattie & Peattie, 2009). At the same time, the field has evolved prolifically with several new conceptualizations and acronyms. Examples include the 4 A’s of marketing, referring to Acceptability, Affordability, Accessibility, and Awareness (Sheth & Sisodia, 2011) and the 4 C’s of marketing, referring to the customer solution, cost, convenience and communication, both emphasising the customer rather than the organisation (Belz & Peattie, 2013; Lauterborn, 1990).

Lunde (2018) noted that marketing theories “evolved from a holistic, macro-level focus to a narrower, micro-level focus” (p. 95) over the years. While in the early years, the theories were used to study society and the environment, later the focus was on studying the practices of companies and institutions as well as the attitudes and behaviours of consumers, which led to a narrowing of theoretical and conceptual ideas and left out other current and future stakeholders. He found that environmental concern models and theories, stakeholder theory, macro/market/institutional systems theories and social psychological theories are the most commonly used, followed by theories such as the dominant social paradigm, Values Beliefs Norms Theory, scepticism theories, Theory of Planned Behavior and Theory of Reasoned Action, which still receive attention in the sustainable marketing literature but are only covered by a few studies (Lunde, 2018). In his view, the emphasis on micro-level and psychological theories has led to fragmented research and inconsistent definitions of sustainable marketing.

To conclude, our study is in line with the observation of prior research that a micro-level and operational focus of sustainable marketing theories and tools on customers and companies is insufficient to address the broader, complex challenges of sustainability, as it ignores complex value relationships and systemic changes required for lasting impact.

## Scoping literature review

Marketing with a focus on sustainability has originated from different areas. Hence, we conduct a scoping review to provide an overview of the topics, concepts and theories being tackled in marketing literature to support a transition towards Marketing in the Anthropocene. A scoping review aims to map the body of literature on a topic area (Arksey & O’Malley, 2005) and perform a qualitative synthesis of the studied literature. Similar to systematic literature reviews, scoping reviews use transparent methods to comprehensively analyse all relevant literature connected to the research question. According to the methodological framework introduced by Arksey and O’Malley (2005), a scoping review comprises five stages: (1) identifying the research question; (2) identifying relevant studies; (3) study selection; (4) charting the data; and (5) collating, summarising, and reporting the results.

Following these steps, in the first stage of this study, we formulated the research questions (see Introduction). The identification of relevant studies included the formulation of formal inclusion criteria (see Table 1) and search strings (see Table 2). The search string was constructed based on the authors' expertise by combining search terms related to concepts of strong sustainability (e.g., flourishing, regeneration, sufficiency, anti-consumption) and sustainable marketing theories (e.g., demarketing, social marketing). The search was performed in the Scopus bibliographic database and in the web-based search engine Google Scholar.

**Table 1:** Formal inclusion criteria of this study

Criterion	Inclusion
Document type	Peer-reviewed journal articles
Time period	Until the end of February 2024
Language	English

**Table 2:** Search strings used in this study

Scopus	TITLE-ABS-KEY (sustainab* AND (sufficien* OR regenerat* OR “net positive” OR handprint* OR slow* OR anti*consum* OR frugal* OR “minimalism”) AND (“social marketing” OR “demarketing” OR “marketing” OR nudg* OR “choice editing”))
Google Scholar	TITLE-ABS-KEY (sustainab* AND (sufficien* OR regenerat* OR “net positive” OR handprint* OR slow* OR anti*consum* OR frugal* OR “minimalism”) AND (“social marketing” OR “demarketing” OR “marketing” OR nudg* OR “choice editing”))

In addition to database screening, we made use of the supplementary search strategies of backward snowballing (i.e., screening reference lists of included studies) and forward snowballing (i.e., screening articles that cite included studies) as well as expert consultations to uncover more relevant articles (Baldassarre et al., 2020; Wohlin, 2014). The study selection stage of the scoping review included multiple steps of paper screening and exclusion with reference to content-related inclusion criteria (see Table 3).

**Table 3:** Content-related inclusion criteria

Conceptual Rigour	Focus on marketing strategies and practices
Impact	Focus on strong sustainability as the outcome

**Fig. 1:**
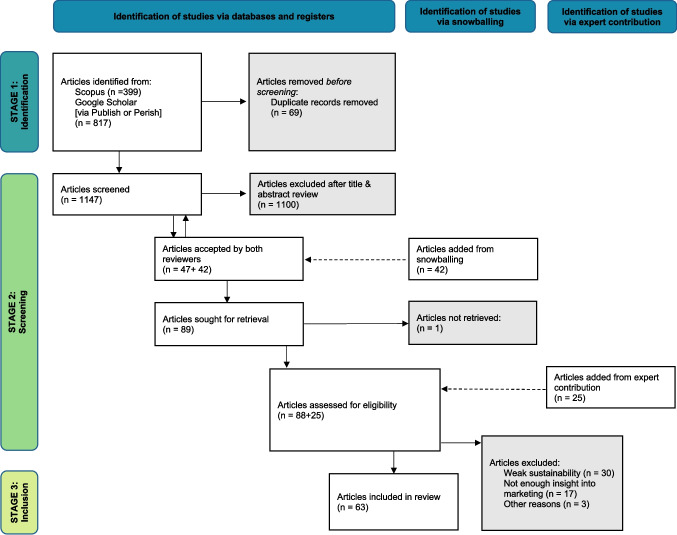
Process of identification of relevant articles. *Developed from *Page et al. (2021)

Figure 1 shows the process of the identification of relevant articles for the study. The database and supplementary searches yielded 1216 publications, with 69 duplicates being removed, resulting in a preliminary sample of 1147 articles. In the first screening step, the titles and abstracts of the articles were thoroughly read. In this phase, two reviewers were tasked with screening. All disagreements as well as ambiguities were resolved in team meetings, which led to the exclusion of 1100 articles. In the second screening step, the two authors focused on the full texts of both the articles that passed the first screening step and those that were identified via snowballing and expert consultation, culminating in a final sample of N = 63.

In the fourth stage of the scoping review, data charting was conducted using qualitative content analysis. The categories chosen for the content analysis aimed to reflect the research questions of this study. The analysis was performed on the full texts of all selected articles using Atlas.ti. The central instrument of analysis constitutes a coding scheme based on a procedure of inductive-deductive coding, which contributes to the intersubjectivity of the procedure, helping others to reconstruct or repeat the analysis. Four of the coders simultaneously coded three initial articles and discussed the coding scheme consisting of the following categories: marketing concepts, marketing theories, marketing instruments, marketing outcomes and examples. Further coding categories were added during the coding process through all coders. Finally, in the fifth stage, the data extracted by means of content analysis was analysed. In the next section of this paper, the outcomes of the study are presented.

## Results

### Overview of articles

**Fig. 2:**
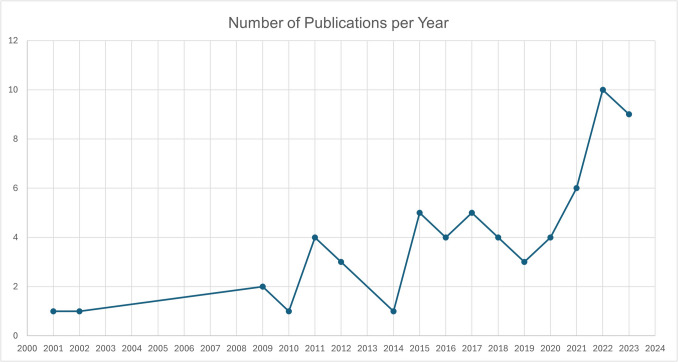
Growing trend of number of articles relevant for Marketing in the Anthropocene

The occurrence of articles that are relevant for Marketing in the Anthropocene has risen significantly in the past 20 years, as visible in Fig. 2. The scoping review was not restricted in the start date, yet the first relevant article was only found in 2001. While the first sustainability marketing research articles already appeared in the 1990s (McDonagh & Prothero, 2014), they did not pass our exclusion criteria, mostly because they held a weak sustainability as opposed to a strong sustainability focus. Another three articles were published until 2010, when the role of marketing in the sustainability crises seems to have become more prominent in research. In the 2010s, 30 articles were published that were relevant to Marketing in the Anthropocene. A further increase since the 2020s can also be observed, with 29 articles published in the span of only four years.

**Fig. 3:**
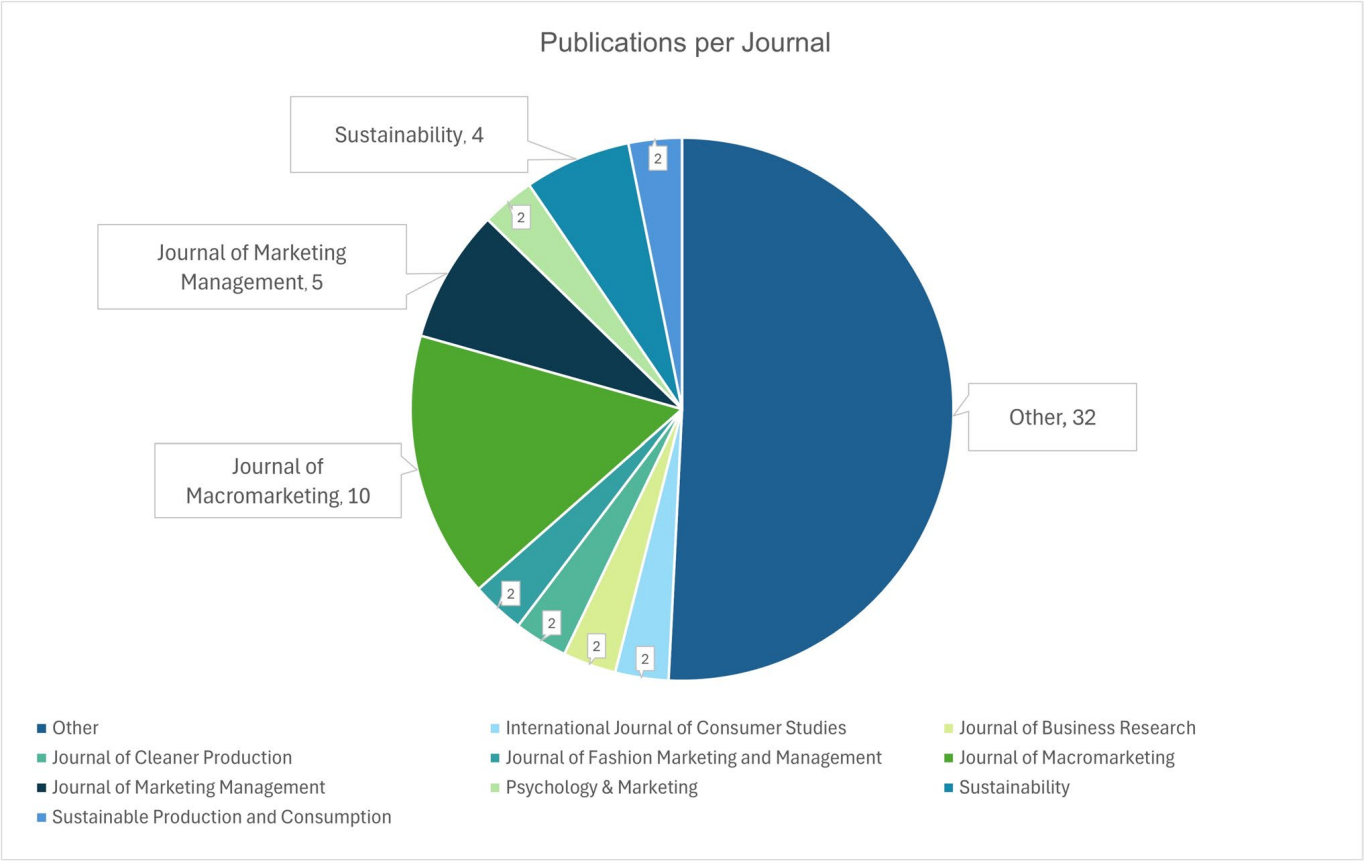
Journal outlets of reviewed articles

Articles that bring an understanding of wider sustainability challenges into the topic of marketing seem dispersed among different journals and disciplines. The 63 articles included in our sample were spread across 41 journals, with 32 of those only featuring one article each (see Fig. 3). The variety of journals publishing research linked to Marketing in the Anthropocene also indicates that the conversation takes place in mainly inter- and multidisciplinary journals. A majority of the articles are published in journals that have a sustainability or interdisciplinary focus. While sustainable marketing already has been a steady topic for years and has had special issues in journals like Academy of Marketing Science (2011), Journal of Marketing, Journal of Marketing Management, Journal of Macromarketing (2010) and Journal of the Academy of Marketing Science, we argue that marketing linked to strong sustainability remained a niche topic in mainstream journals and was mainly covered in sustainability-focused or interdisciplinary outlets. In our search, for instance, the Journal of Macromarketing dominates with ten articles. Macromarketing takes a multidisciplinary perspective as it analyses the impact of marketing on wider society and economy and has a long tradition of addressing topics around marketing and the environment (Fisk, 1973). Similarly, interdisciplinary journals such as Psychology & Marketing and Sustainability show several publications related to the topic.

Articles in the sample used some recurrent themes to explain the need for a different practice of marketing. Most of the reviewed articles refer to sustainability challenges caused by the Anthropocene, such as climate change and its impacts, but also social challenges, including poverty and injustice. Another common theme was the societal context in which marketing takes place. Many of the publications referred to the “consumerist” society and culture. Consumption in this society and culture is linked to wellbeing and happiness. This is also identified as a barrier to promoting sufficiency through alternative marketing concepts. Similarly, many of the articles refer to the economic context that drives mainstream marketing. Economic forces of influence are the capitalist economy and the societal expectation of, and dependency on economic growth. This growth paradigm usually entails a sales orientation on the side of businesses and is therefore identified as a barrier to alternative marketing approaches. The role of marketing was also discussed by several of the papers. There were two different roles presented: on the one hand, the role of marketing as “traditionally focused on creating demand for a product and service” (Armstrong Soule & Reich, 2015, p. 1404). On the other hand, articles also described the potential role that marketing could play to promote strong sustainability, for instance stating that “the marketing discipline is in an immediate position to initiate change as it has the means to bring about radical transformations in consumption patterns” (Kelleci & Yildiz, 2021, p. 11). This reiterates this paper’s call for marketers to take responsibility and use their influence to adopt a practice of Marketing in the Anthropocene.

Articles were selected based on a strong understanding of sustainability. Therefore, they largely applied concepts of marketing that went beyond weak sustainability (i.e., “green”) marketing. The most commonly referred to alternative marketing concept was that of social marketing, found in 30 of the reviewed articles. Not all of those articles used the concept, some also merely introduced it in the literature review or discussion. The second most commonly referred-to concept was demarketing, mentioned by 20 papers. In nine papers, the concept was specified to green demarketing, which means discouraging consumption for environmental reasons. These three concepts were often linked or used interchangeably. The papers by Balderjahn & Hoffmann (2023) and Balderjahn and Appenfeller (2023), for instance, consider (green) demarketing to be a subsection of social marketing approaches. Another commonly referred-to concept was sufficiency-promoting marketing, mentioned in eight publications. It can be observed that the marketing concepts of (green) demarketing and sufficiency-promoting marketing aim to promote strong sustainability by reducing consumption levels. Beyond issues of consumption, the concept of social marketing was applied more broadly and often linked to promoting human health. Other alternative concepts were ethical marketing, degrowth-oriented marketing, transformative sustainability marketing and socially responsible marketing.

### Marketing outcomes

One of the themes focused on in the analysis was the question of which outcomes or impacts of marketing were mentioned in the publications. Two distinct sets of outcomes could be identified: on the one hand, the (positive and negative) effects of the mainstream marketing practices mostly in place, and on the other hand, the (positive and negative) outcomes of what we term Marketing in the Anthropocene. Table 4 graphically represents the outcomes that were commonly referred to for the two types of marketing and sometimes present in both.

**Table 4:** Marketing outcomes in reviewed articles

Outcomes	Details (& exemplary reference)	Mainstream Marketing/ Marketing in the Anthropocene?
**Positive outcomes**		
Anti-consumption and reduced consumption	• Lower consumption of resources or intentionally refused consumption of resources as a response to overconsumption (Hwang et al., 2016)	Marketing in the Anthropocene
Sufficiency	• Sufficiency-oriented lifestyles or strategies to have consumption levels that are “adequate but not wasteful” (Balderjahn & Hoffmann, 2023, p. 3)	Marketing in the Anthropocene
Wellbeing	• Higher life satisfaction with rising economic growth; well-being through consumption; e.g., “shopping well-being” and identity construction (Ertekin & Atik, 2015)	Mainstream marketing
	• Increased well-being as some constraints of a consumer culture are lifted, e.g., higher equality, personal quality of life, more self-determination; Also increased well being through better health, for instance through social marketing against tobacco (Seegebarth et al., 2016)	Marketing in the Anthropocene
Positive consumer perception	• Perception of trustworthiness and quality of a brand (Ramirez et al., 2017) • Emotional connection of consumer to the brand (Pham et al., 2023)	Marketing in the Anthropocene
Sustainable consumption	• Consuming products or services that are environmentally and/or socially sustainable (Sodhi, 2011) • Consuming at a level that is sustainable (e.g., mindful consumption) (Gossen et al., 2019)	Both
Rebound effect	• Money that is saved by foregoing consumption can be used to further reduce resource use (e.g., environmental cause donation) (Balderjahn & Hoffmann, 2023)	Marketing in the Anthropocene
Regeneration	• Slower or reduced consumption can lead to the natural regeneration of resources (Ertekin & Atik, 2015) • Fairer labour conditions for workers and potential for decreased work hours (Haider et al., 2022) • Developing non-material values, such as art, music and care economy (Haider et al., 2022)	Marketing in the Anthropocene
**Negative outcomes**		
Overconsumption	• Environmentally: Consuming resources to an extent that the environment cannot recuperate (Seegebarth et al., 2016) • Socially: High consumption levels lead to poverty, inequality, a culture driving people to consume and potentially into debt, negative impacts on wellbeing (Ertekin & Atik, 2015) • Overconsumption is usually linked to wealthy nations but increasingly to other countries that gain prosperity (Ardley & May, 2020)	Mainstream marketing
Lack of wellbeing	• Negative impacts on wellbeing due to the culture of consumption exacerbating inequalities and poverty; lower quality of life (Ardley & May, 2020)	Mainstream marketing
	• Involuntary consumption reduction (e.g., through poverty) leads to reduced wellbeing (Gorge et al., 2015)	Marketing in the Anthropocene
Negative consumer perception	• Consumer perception that business and marketing are to blame for overconsumption (rather than consumers) (Pereira Heath & Chatzidakis, 2012) • Perception of untrustworthiness and greenwashing; this is often linked to the reputation of the sender (Armstrong Soule & Reich, 2015) • Concerns about product quality of “green” products (Ramirez et al., 2017)	Both
	• Perception of poor labour and social practices (Pinto & Casais, 2023)	Mainstream marketing
Sustainable consumption as a distraction	• Focusing on the production and consumption of sustainable or “greener” products and services can distract from the need to reduce overconsumption volumes (Ardley & May, 2020)	Mainstream marketing
Rebound effect	• Money saved from foregoing a purchase is used to consume other, additional goods (Balderjahn & Appenfeller, 2023) • Demarketing messages may entice disagreeing consumers to buy more (Yakobovitch & Grinstein, 2016) • Moral licence: Consumers feel they act sustainably, so they can act less sustainably in some situations (Balderjahn & Appenfeller, 2023)	Marketing in the Anthropocene

While the publications reviewed argued for a change away from mainstream marketing, they were also nuanced in their appraisal of potential negative side effects of alternative approaches such as demarketing and sufficiency-promoting marketing. They also acknowledged the potential connection between economic wellbeing (economic growth) and quality of life that builds the rationale for continuing with the status quo.

### Marketing tools and instruments

To help marketers and marketing researchers develop a new form of marketing, the literature review also identified marketing tools and instruments for a strongly sustainable practice that have been used or have been suggested by research. Table 5 details the tools identified in the reviewed papers. They are structured alphabetically with a short explanatory description. All of these tools are already applied in marketing practice and some are additionally applied in research, for instance when the 4P marketing mix is used to structure findings or when communication messages are used in an experiment to determine customer responses.

**Table 5:** Key marketing tools found in reviewed articles. Note: tools with an asterisk (*) were only mentioned in one article but are included for potential interest for Marketing in the Anthropocene

Tool	Description	Exemplary reference
4 (or 5) Ps Marketing Mix	Product, Price, Place, Promotion, (People)	Lawrence and Mekoth (2023)
4 As*	Alternative marketing mix developed by Sheth & Sisodia (2011): Acceptability, Affordability, Accessibility, Awareness	Varey (2012)
Ban	Decision by a producer, supplier or government to not allow the sale of a certain product or service, for instance because of its unsustainability	Bocken (2017)
Boycott	Action of intentionally foregoing consumption of a product or service. Examples could be consumers boycotting a brand or brands boycotting price reductions on Black Friday	Seegebarth et al. (2016)
Business model (canvas)	Changes to a business’ value proposition, creation, delivery and capture to offer a sustainable product or service, for example offering repair or reuse services The business model canvas (Osterwalder & Pigneur, 2010) is one example of a tool to support business model innovation	Kelleci & Yilidiz (2021)
Choice editing	Similar to nudging, choice editing means creating an infrastructure in which some choices are easier or more obvious to consumers, for instance the sustainable choice	Bocken and Allwood (2012)
Communication messages	Message framing to stakeholders, including customers but also staff members, etc. Examples might be advertisement campaigns	Hall (2018)
Durability	It is about promoting long product lifetimes and extended use through product-service systems, reuse or repair can remove the need to purchase new products Durability as a product property is part of the 4Ps but is specifically added because of its frequent mentioning	Gossen et al. (2019)
Education	Information and knowledge sharing about the impact of (unsustainable) consumption and sustainable alternatives	Peattie and Peattie (2009)
Feedback	Providing feedback to customers, for instance on their consumption or the impact of their consumption, might help to raise awareness and reduce consumption levels	Kollmus & Agyeman (2002)
Framing	Using a frame to positively or negatively position a message that might then be received differently by the audience	Kim et al. (2018)
Labelling	Providing information on products, such as ecoscores or carbon labels, to raise awareness and potentially influence consumption choices	Majer et al. (2022)
Nudging	Small-scale interventions, for instance through design choices, to guide consumers to choose the sustainable option	Sharma and Silal (2023)
Pricing	Altering the price point and pricing structure can reduce consumption (e.g., by premium pricing). This may be problematic for accessibility	Raab et al. (2023)
Segmentation	Identifying and targeting customer segments to ensure that the marketing message or offer is received as intended. For instance, anti-consumption messages are received differently by people from different income levels	Lim (2016)
Social marketing mix*	Alternative marketing mix, suggested for instance by Peattie et al. (2009): Social propositions, Accessibility, Social costs of involvement, Social communication	Lowe et al. (2015)
Storytelling	Using storytelling techniques to make information more engaging, for instance reporting about the experiences of other customers with (anti-) consumption	Abu Bakar et al. (2021)

### Marketing theories

The call for Marketing in the Anthropocene is linked to a need to review current theories in marketing in terms of their suitability and to build knowledge for a future of strongly sustainable marketing. This is in line with Lim (2016) who states that “[t]he multitudes of theories and practices in marketing are constantly evolving to address new trends and events in the marketplace, both locally and globally” (p. 237). The overarching trends of the Anthropocene and its sustainability challenges therefore warrant a review of marketing theories that can be most insightful for future research. In standard sustainability marketing, the most popular theories include values and societal theories (e.g., dominant social paradigm, Value-Beliefs-Norms Theory), environmental theories (e.g., environmental concern theories), business and institutional theories (e.g., stakeholder theory, resource-based view), consumer and behavioural theories (e.g., Theory of Planned Behaviour, Theory of Reasoned Action) and socio-psychological theories (e.g., Construal Level Theory) (Lunde, 2018). Some of these theories are borrowed by publications relevant to Marketing in the Anthropocene. Table 6 shows the theories already applied and further theories that might be of use. The list shows a wide range of disciplines, ranging from more standard marketing, over psychological and behavioural, to economic theories. They also cover a range of levels from micro, over meso to macro foci, with some theories, like the Theory of Planned Behavior, suited to understand individual behaviour, while others, like the dominant social paradigm, trace and explain society-wide patterns. To note, the 4P marketing mix is also listed in the section on tools above. It is used by marketers to plan the product, place, promotion, and price of a marketing campaign.

**Table 6:** Marketing theories identified in reviewed articles (exemplary reference in brackets)

Theories applied in Marketing in the Anthropocene (* for theories applied by more than one source)	• 4 P Marketing Mix* (Gossen & Kropfeld, 2022) • Appraisal-emotional response-coping framework (Ramirez et al., 2017) • Attribution Theory* (Reich & Soule, 2016) • Commitment Theory (Raab et al., 2023) • Consumer Value Creation (Jutbring, 2018) • Dominant Social Paradigm* (Gorge et al., 2015) • Elaboration Likelihood Model (Abu Bakar et al., 2021) • GREEN framework of sustainable marketing (Lunde, 2018) • Habits (Armstrong Soule & Reich, 2015) • Institutional Theory (Ertekin & Atik, 2015) • Multi-level perspective (Little et al., 2019) • Personal norms (Frick et al., 2020) • Pro-social and pro-environmental behaviour* (Kim et al., 2018) • Protective Motivation Model (Balderjahn & Hoffmann, 2023) • Signalling (Sekhon & Armstrong Soule, 2020) • Sustainable Consumption Strategies framework (Bocken, 2017) • Sustainability-Rooted Anti-Consumption (Seegebarth et al., 2016) • Social norms* (Balderjahn & Appenfeller, 2023) • Social Practice Theory* (Little et al., 2019) • Stimulus-organism-response theory (Mohamed Sadom et al., 2020) • The five dimensions of sustainability marketing (Lim, 2017) • Theory of Planned Behavior* (Lowe et al., 2015) • Theory of Reasoned Action* (Raab et al., 2023) • Theory of Reciprocity (Lowe et al., 2015) • Theory of Structuration (Ardley & May, 2020) • Theory of Trying (Armstrong Soule & Reich, 2015)
Additional theories suggested/ discussed for Marketing in the Anthropocene	• 4 Fundamental Explanada of Marketing (Lunde, 2018) • Agency Theory (Connelly et al., 2011) • Altruism Theory (Kollmuss & Agyeman, 2002) • Commons Resolution Framework (Rashidi-Sabet & Madhavaram, 2022) • Construal level theory (Lawrence & Mekoth, 2023) • Consumer Behavior Theory (Varey, 2012) • Cultural Theory (García-de-Frutos et al., 2018) • Descriptive norms (Yakobovitch & Grinstein, 2016) • Discourse Theory (García-de-Frutos et al., 2018) • Framing (Niessen et al., 2023) • Maslow’s Hierarchy of Needs (Varey, 2010) • Norm Activation Model (Majer et al., 2022) • Organizational ecology (Connelly et al., 2011) • Political economy (Lloveras et al., 2022) • Prospect Theory (Lawrence & Mekoth, 2023) • Reason Theory Perspective (Seegebarth et al., 2016) • Regulatory Focus Theory (Lunde, 2018) • Relationship marketing (Kamila & Jasrotia, 2023) • Resource dependence theory (Connelly et al., 2011) • Responsible consumption theory (Sodhi, 2011) • Self-determination Theory (Solér, 2012) • Social Network Theory (Peattie et al., 2009) • Theory of Consumption Values (Lawrence & Mekoth, 2023) • Theory of Exchange (Lunde, 2018) • Transaction Cost Economics (Connelly et al., 2011) • Upper Echelons Theory (Connelly et al., 2011) • Value-Belief-Norm (Haider et al., 2022)

The list is too long to discuss all theories at length, so we only briefly introduce the three most frequently used theories. First, attribution theory is applied by three publications: Hwang et al. (2016), Reich and Armstrong Soule (2016) and Raab et al. (2023). Attribution theory suggests that individuals try to legitimise their own behaviour by attributing it to others. In the research mentioned, attribution theory was used to assess whether research participants find a source of anti-consumption messages genuine or trustworthy and who they think is responsible for sustainable behaviour. Second, the concept of social norms is applied by several of the publications: Balderjahn and Appenfeller (2023), Balderjahn and Hoffmann (2023), Frick et al. (2020), Frick et al. (2021), and Lowe et al. (2015). These papers base their research on the potential role of social norms on promoting reduced resource consumption. Social norms can be understood as a moral imperative or as the expectation of “how other persons commonly behave or consume” (Balderjahn & Appenfeller, 2023, p. 3). Different types of social norms may influence how consumers respond to alternative marketing such as anti-consumption appeals. In the papers reviewed, the concept is also used together with others, such as personal norms or the Theory of Planned Behavior. Third, the Theory of Planned Behavior is applied in four papers: Armstrong Soule and Reich (2015), Abu Bakar et al. (2021), Lowe et al. (2015) and Raab et al. (2023). This theory argues that behavioural intention can be inferred from an individual’s attitude, subjective norms and perceived behavioural control. The studies applying this theory were trying to understand consumers’ intended behaviour change towards sustainability.

## Discussion

Based on the findings from the scoping literature review, in this section we first reflect on the different eras in which sustainability issues started to influence marketing, followed by a framework bridging tools and theories towards Marketing in the Anthropocene.

### Towards the era of Marketing in the Anthropocene

**Fig. 4:**
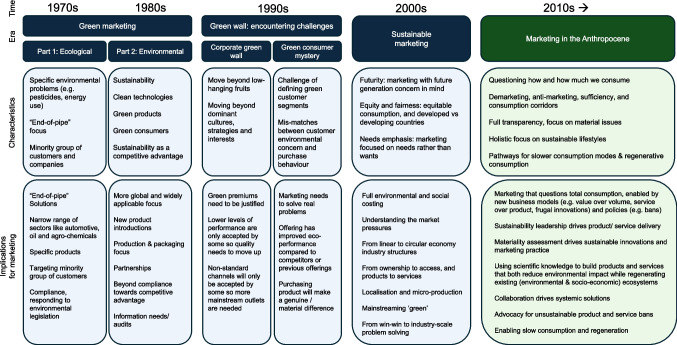
The eras towards Marketing in the Anthropocene. *Note. Eras developed from *Peattie (2001)*, plus insight from the present study on Marketing in the Anthropocene*

The developments of bringing sustainability into marketing research and practice may be divided into four main eras: it starts with the era of green marketing in the 1970s and 1980s, followed by the 1990s where a 'green wall' is hit by companies, and sustainable marketing in the 2000s where sustainability concepts such as equity, and fairness as well as “needs rather than wants” emerge in marketing (see Peattie, 2001). We argue for the advent of a new era from the 2010s onwards: the era of Marketing in the Anthropocene (see Fig. 4).

The 1970s sub-era of *ecological marketing* was marked by tackling specific ecological problems such as pesticides and energy use. It was characterised by an 'end of pipe' focus, targeting issues like waste rather than holistically changing product design (see Peattie, 2001). The target sectors and customers were fairly limited, including sectors like automotive and agro-chemicals, while focusing on the 'greenest' customers mainly. The following 1980s sub-era of *environmental marketing* was marked by a more holistic focus by adopting green technologies and design and focusing on customers who are concerned about a wider set of sustainability issues such as health, animal cruelty, over-packaging, and shortening product life spans (Peattie, 2001). Sustainability started to be seen as a potential source of competitive advantage. It was argued that (environmental) legislation would increase innovation and that win–win solutions could be created that would simultaneously improve environmental and economic performance by saving costs through efficiency gains, and winning over new customer groups (e.g., Porter & van der Linde, 1995). In the 1990s, as customer awareness of sustainability issues grew, so did awareness of greenwashing and untrue or exaggerated claims (Peattie, 2001). Companies needed to make changes beyond the low hanging fruits of efficiencies, that saved cost and resources simultaneously, to make a bigger impact. Moreover, while customers showed interest in sustainability issues, this did not proportionally translate into increased sustainable purchases (Kalafatis et al., 1999; Peattie, 2001). Hence, Peattie (2001) refers to the era of the *'green wall'* in the 1990s where companies experienced a more challenging sustainability market. Interestingly, from a research perspective, McDonagh and Prothero (2014) noted that while companies started to embrace sustainability in marketing practice in the late 1990s, the topic did not become widely embraced by mainstream marketing journals. Simultaneously, NGOs started targeting greenwashing, engaging in a deeper scrutinization of corporate marketing tactics. This would have been the ideal time for mainstream marketing researchers to shift their focus towards sustainable marketing, but a grand shift did not happen.

The work by Peattie (2001) ends with the era of "*sustainable marketing*" (the 2000s) which is a prelude to what we refer to as “Marketing in the Anthropocene.” While definitions vary greatly, sustainable marketing conceptually builds on the notion of sustainable development (Brundtland, 1987), including greater awareness of the implications of marketing for the future and effects on others, including equity and fairness considerations. This era is characterised by full environmental and social cost accounting, a focus on needs rather than wants, an increasing awareness of closing material loops, and the localisation of production and the transformation of business models (e.g., from products to services) (Peattie, 2001). Rather than looking for individual win–win situations, industry collaborations are developed to tackle sustainability issues collaboratively (Porter & van der Linde, 1995).

As for the other eras, there are also no hard lines between the latter observed eras, and we have not fully embraced the potential of sustainable marketing yet (e.g., full accounting of societal and environmental issues). Hence, we propose the era of *Marketing in the Anthropocene* as an inspirational, forward-looking concept, tool and practice for marketers and marketing researchers.

We define Marketing in the Anthropocene as follows:

*A marketing practice and concept that recognises the ecological limits of the planet, impacts of business on nature and society, and the responsibility marketers hold in steering towards a strongly sustainable future. Realising the detriment of overconsumption, it advocates for consumption within planetary limits, reducing unnecessary consumption and focusing on meeting needs. It also works towards improving social and environmental wellbeing through regeneration.*

The era of Marketing in the Anthropocene builds on and expands sustainable marketing which started to create building blocks such as a future focus, a shift from wants to needs, and ethical topics such as equity and fairness. Based on our analysis, the era of Marketing in the Anthropocene could have conceptually started in the 2010s when the number of articles on forward-looking topics like demarketing, consumption reduction, and sustainable lifestyles increased.

Based on the articles reviewed, Marketing in the Anthropocene must go against the current levels of overconsumption, in particular in the Global North. The dominant social paradigm of consumerism and economic growth through increased sales is reinforced by mainstream marketing practice and needs to be countered by a wave of demarketing, social marketing and sufficiency-promoting marketing to rectify the perception that current consumption levels are normal. Marketing can also help influence the social and cultural perceptions of wellbeing, promoting a focus on immaterial values, such as community, culture and care. Based on its previous role in promoting increasing consumption, marketing practice can take up a prominent role in supporting sufficiency. On a larger scale, changes to the economic system have also been suggested by degrowth and post-growth scholars. They argue that the economy itself needs to be refocused away from an obsession with economic growth towards being ecologically sustainable and socially desirable (Nesterova, 2020). Degrowth takes this starting point to highlight the need for a deliberate reduction of economic activity, with a particular focus on degrowing harmful sectors (Savin & van den Bergh, 2024). This is in line with Marketing in the Anthropocene, where unnecessary consumption is halted. Yet, degrowth and its definitions are still debated, as are the ways to reach a post-growth or steady state economy (Savin & van den Bergh, 2024).

In addition to limiting negative impacts, marketing can also play a role in *improving* existing systems through “regenerative consumption,” a term still largely undefined, yet promising. Regenerative consumption may involve the use of goods and services that have a net positive impact on nature and society, to directly or indirectly change the world in a way that is good for natural ecosystems and people (Hahn & Tampe, 2021). For example, buying single-origin coffee from smallholder farmers that use regenerative agriculture can support historically disadvantaged communities, and incentivise stewardship practices to restore degraded forests. Such consumption aims to fulfil an ecosystem function and to contribute to the health of an ecosystem rather than damaging it.

Indigenous communities already practice regeneration to a large extent: Oakes (2018) has reported that Alaskan indigenous communities prefer to use the forests for economic activities in a symbiotic way that incentivises their protection instead of just creating natural parks. This goes further than preservation strategies that often exclude indigenous forest management, aiming to simply reduce harm by “preserving” things as they are, rather than actively improving the health of ecosystems (Stevens, 2014). Part of this is a philosophical shift: from a worldview of natural ecosystems simply serving as a source of raw materials for building the economy, towards a view where humans and the economies they have built are an embedded part of a larger socio-ecological ecosystem (Folke et al., 2010). Research has only recently started to embrace indigenous knowledge to create regenerative business practices (Konietzko et al., 2023). This is still new territory with limited research, besides for instance references on regenerative practices in research on food marketing (e.g., Milani et al., 2017; Solér, 2012). While marketing is not a core theme in this existing research, understanding more deeply how environmental regeneration may be conducted in an economically viable way that supports communities while improving the health of ecosystems, and exploring how this shift in business can be communicated to customers, can be the start of reimagining a more regenerative approach to marketing.

### Bridging tools and theories for Marketing in the Anthropocene

*“The Good Anthropocene has generated better lives for billions of ordinary humans, for the first time in human history [...] But there is also a Bad Anthropocene. The Bad Anthropocene consists of the many changes that threaten the achievements of the Good Anthropocene [...] Can we preserve the best of the Good Anthropocene and avoid the dangers of the Bad Anthropocene?”*

-David Christian (2018, p. 303)

**Fig. 5:**
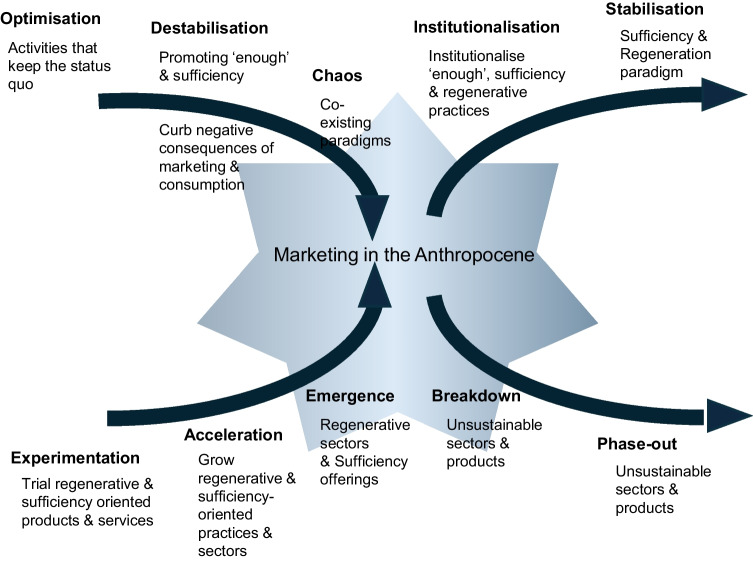
**X-curve with illustrative examples for Marketing in the Anthropocene.** Source: Building on Hebinck et al. (2022)

Marketing in the Anthropocene means that parts of the old system need to be dismantled while other parts need to be (re)built. This is in line with the idea of an X-curve in Transitions Research, which is a simplified depiction of a transition that captures both patterns of build-up (upward sloping line of the X) and breakdown (downward sloping line of the X), as well as the interactions between these developments (Hebinck et al., 2022). Figure 5 presents the X-Curve with illustrative examples for Marketing in the Anthropocene showing which activities will need to be experimented with and built up, and which ones ‘broken down.’ Examples are derived from Table 7, explained next.

**Table 7:** A blueprint for Marketing in the Anthropocene. *Note: Building on *Tables 5 & 6

**Dismantling the Bad Anthropocene**	
**Strategies**	Possible marketing theories, tools and instruments
Promoting sufficiency	Theories targeting needs vs wants: • Maslow's Hierarchy of Needs (Varey, 2010) Theories focusing on norms and values: • Personal norms/ Social norms/Descriptive norms/ Value-Belief-Norm/Norm Activation Model (Frick et al., 2020) • Altruism theory (Kollmuss & Agyeman, 2002) Theories targeting practices and behaviour: • Attribution theory (Reich & Armstrong Soule, 2016) • Social Practice Theory (Little et al., 2019) • Pro-social and pro-environmental behaviour (Kim et al., 2018) • Theory of planned behaviour (Lowe et al., 2015) Generic tools and instruments: • Communication messages (Hall, 2018) • Education (Peattie & Peattie, 2009) • Feedback (Kollmuss & Agyeman, 2002) • Labelling (Majer et al., 2022) • Nudging (Sharma & Silal, 2023) • Social marketing mix (Lowe et al., 2015) • Storytelling (Abu Bakar et al., 2021) • Segmentation (Lim, 2017)
Phasing out sectors/ products	Theories focused on transitions: • Institutional Theory (Ertekin & Atik, 2015) • Multi-level perspective (Little et al., 2019) • Political economy (Lloveras et al., 2022) • Cultural theory (García-de-Frutos et al., 2018) • Dominant Social Paradigm (Gorge et al., 2015) Theories focused on communication and education: • Discourse Theory (García-de-Frutos et al., 2018) • Framing (Niessen et al., 2023) Generic tools and instruments: • Education (Peattie & Peattie, 2009) • Bans (Bocken, 2017) • Boycotts (Seegebarth et al., 2016)
Avoiding negative side effects of marketing and consumption	Theories focused on communication and education: • Discourse Theory (García-de-Frutos et al., 2018) • Framing (Niessen et al., 2023) Generic tools and instruments: • Education (Peattie & Peattie, 2009) • Feedback (Kollmuss & Agyeman, 2002) • 4 P's, e.g.: Durability (product longevity) (Lawrence & Mekoth, 2023) • Nudging (Sharma & Silal, 2023)
**Enhancing and building the Good Anthropocene**	
**Strategies**	Possible marketing theories, tools and instruments
Promoting new regenerative products and services	Theories promoting new products and services • 4 Ps (marketing mix) or 4As alternative marketing mix (Gossen & Kropfeld, 2022) Generic tools and instruments: • 4 Ps (marketing mix) (Lawrence & Mekoth, 2023) • 4 As (alternative marketing mix) (Varey, 2012) • Education (Peattie & Peattie, 2009) • Framing (Kim et al., 2018) • Labelling (Majer et al., 2022) • Segmentation (Lim, 2017) • Social marketing mix (Lowe et al., 2015) • Storytelling (Abu Bakar et al., 2021)
Encouraging regenerative practices	Theories promoting new behaviours, needs and wants: • Habits theory (Armstrong Soule & Reich, 2015) • Maslow’s Hierarchy of Needs (Varey, 2010) • Attribution theory (Reich & Armstrong Soule, 2016) Generic tools and instruments: • 4 Ps (marketing mix) (Lawrence & Mekoth, 2023) • 4 As (alternative marketing mix) (Varey, 2012) • Education (Peattie & Peattie, 2009) • Framing (Kim et al., 2018) • Nudging (Sharma & Silal, 2023) • Storytelling (Abu Bakar et al., 2021)
Promoting new regenerative sectors	Theories promoting the “new normal”: • Personal norms/ Social norms/Descriptive norms/ Value-Belief-Norm/Norm Activation Model (Frick et al., 2020) • Habits theory (Armstrong Soule & Reich, 2015) • Maslow’s Hierarchy of Needs (Varey, 2010) Generic tools and instruments: • 4 Ps (marketing mix) (Lawrence & Mekoth, 2023) • 4 As (alternative marketing mix) (Varey, 2012) • Education (Peattie & Peattie, 2009) • Framing (Kim et al., 2018) • Storytelling (Abu Bakar et al., 2021)

The blueprint proposed in Table 7 includes potential theories, marketing tools and instruments from our study for dismantling aspects of the Bad Anthropocene and encouraging aspects of the Good Anthropocene. Articles relevant for dismantling the Bad Anthropocene refer to topics like promoting sufficiency (Gossen et al., 2019), phasing out harming products and sectors (Bocken & Allwood, 2012), as well as avoiding negative consequences like boomerang or rebound effects (Yakobovitch & Grinstein, 2016). As for the build up of a new normal, this is where regenerative practices, products and practices come in (Konietzko et al., 2023).

To start, the majority of our reviewed articles identified tools, methods and theories that could support the dismantling of the unsustainable system, including areas like demarketing and sufficiency. Simpler lifestyles could be made more appealing (Seegebarth et al., 2016) building on the tools, instruments and theories of marketing, e.g., by showcasing sufficiency behaviour via social media and making it an important part of a brand (Gossen & Kropfeld, 2022; Sekhon & Armstrong Soule, 2020).

However, next to the strategy of 'less' we also need to have a viewpoint of what we need more of: a positive outlook on lifestyles which is exciting, interesting, and maybe even aspirational. Regenerative practices and products come in here (Hahn & Tampe, 2021); so could the idea of flourishing (Ehrenfeld & Hoffman, 2013). Yet, the work on regenerative practices and certainly regenerative marketing is still untapped territory, although it is clear that we need to start identifying and building up new practices, products, services and even sectors that contribute to nature regeneration, societal and personal wellbeing (Seegebarth et al., 2016). Marketing could contribute to regeneration by pushing values such as true pricing and transparent supply chains, informing consumer choice and behaviour in a way that promotes regeneration (Konietzko et al., 2023). The promotion of lifestyles that help consumers to fulfil ecosystem functions could be the next step. In adjacent literature on calculating the impact of lifestyles, Druckman & Jackson (2010) propose going back to the bare necessities to understand what we really need, and Druckman and Gatersleben (2019) follow with proposals to undertake low carbon leisure time activities that contribute to wellbeing, rather than materialistic consumption that might actually make people unhappier. Bridging knowledge on the impacts of activities with possible positive pathways forward for sustainable lifestyles can feed into the nurturing of the new concept of Marketing for the Anthropocene.

### The pathway towards marketing in the anthropocene

The pathway towards Marketing in the Anthropocene is not straightforward and needs deliberate and persistent change from key actors involved and entails some key considerations. First, sufficiency and regeneration are two sides of the same coin: a sustainable lifestyle would most likely combine aspects of reduced consumption with regenerative practices and consumption to make the remaining consumption more positive and/ or with less negative impact. Second, to normalise both concepts, a large cultural transformation would be needed at the level of institutions (norms, values, culture, legislation) (Scott, 2005). Research has linked materialism to unsustainable lifestyles, and the value of materialism may be embedded more in some cultures than in others (Yakobovitch & Grinstein, 2016). Solutions thus need to take into account dominant cultures, and paint different pictures for future sufficiency and regenerative lifestyles supported by marketing practices that discourage old and promote new behaviours. Third, social equity and justice also need to be considered when reassessing marketing tactics, in particular when marketing affects the realignment of global value chains. Fourth, regeneration and sufficiency go back to often forgotten times and practices (see Konietzko et al., 2023; Niessen et al., 2023), but in a globalised modern economy, it is essential to start interpreting these concepts in novel ways to develop pathways to preserve the good parts of the Anthropocene and dismantle the bad parts of the Anthropocene. Modern concepts like digitalisation and the advent of AI (Nishant et al., 2020; Kar et al., 2022) and the service economy (see e.g. Stahel, 2008; Tukker, 2015) need to be leveraged, as introduced in Table 8 as cross-cutting themes. Table 8 is a mere starting point to open up this discussion, including guiding questions for the development of the research and practice of Marketing in the Anthropocene.

**Table 8:** Future guiding topics and questions for research and practice. Note: An asterisk (*) means that the concept is derived from literature beyond our sample (literature provided in brackets)

**Dismantling the Bad Anthropocene**	
**Instruments/ strategies**	**Guiding questions**
Promoting sufficiency	• How can we formulate lower and upper boundaries for needs and consumption patterns (consumption/ sufficiency corridors)? • How can marketing tactics help normalise sufficiency-oriented behaviour within the corridors? • How can marketing help make sufficiency-oriented behaviour attractive?
Phasing out sectors/ products/ practices	• How can marketing be used for e.g. labelling to phase out the worst products? • Which nudging or social marketing techniques may be used to drive people away from ‘unsustainable sectors’ like fossil fuels and meat production? • How can product bans be made acceptable by using tools and techniques from marketing?
Avoiding unintended consequences	• What are the boomerang effects, rebound effects, and unintended consequences more generally of marketing? • How can we use marketing (such as informing/ educating and nudging) to encourage desired behaviours?
**Enhancing and building the Good Anthropocene**	
**Instruments/ strategies**	**Guiding questions**
Promoting new regenerative sectors	• What are ‘net positive’ or regenerative sectors? How can they be nurtured? • What could new sectors like regenerative Food and Agriculture (Rhodes, 2017), Regenerative Built Environment (du Plessis & Brandon, 2015) and regenerative consumer goods (Gualandris et al., 2024) look like and how might marketing help create these novel sectors?
Regenerative practices, products and services	• What are regenerative practices, design, production processes and supply chains? (e.g., Gualandris et al., 2024) • How can marketing help create and promote these?
Macro–perspective marketing for all marketeers	• How can we take a more holistic view of the role of marketing in society? • How can we bring in a more holistic macro-marketing perspective into all marketing? • How can marketing practice start focusing on what is really needed, and focus on benign and regenerative products and services?
**Cross-cutting themes from adjacent marketing literature**	
**Instruments**	**Guiding questions**
Twin transition of digital & sustainable economy*	• How can we leverage digitalisation for a sustainable transition while minimising its footprint? (e.g., Bohnsack et al., 2022) • How can AI help ‘dematerialise’ consumption and reduce the overall footprint? (e.g., ​​Paredes-Frigolett & Pyka, 2023) • How can we leverage social media platforms and other forms of digitalisation to normalise sustainable lifestyles in the Anthropocene? • How can we instil the prudent use of digitalisation and take on strong sustainability requirements for digitalisation? (e.g., Bohnsack et al., 2022) • How can we encourage sustainable usage of digitalisation and social media (& discourage unsustainable usage) and move away from addictive algorithms towards new forms of sustainable marketing that fit in the Good Anthropocene? (e.g., Bocken & Short, 2021)
Service economy*	• How might service driven business models help create regenerative offerings? What new products and services may be imagined and put forward by marketers? (e.g., Konietzko et al., 2023) • What novel sustainable services & service business models might be imagined that include services like long warranties, spare parts availability, and right to repair? (see e.g., Circular Economy Package as part of the Green Deal) (e.g., Tukker, 2015)
Social equity and justice*	• What role do marketing practices play in addressing (global) social equity and justice? (e.g., Hornik & Rachamim, 2023) • How do sustainability marketing initiatives impact marginalised communities, and how can they be designed to empower these communities? (e.g., Das & Bocken, 2024)

### Insights for future practice

Marketing is a driving force in business but obviously has its limits in transforming lifestyles of individuals holistically. Marketing communication does play an important role in properly portraying a company's commitment to Anthropocene issues. Success depends on the company consistently delivering what it promises. Ethical concerns arise when companies engage in greenwashing or make unsubstantiated claims and unintended social and environmental consequences are not accounted for. In terms of social impact, increasing consumption brings higher profits to companies, while higher profit margins charged to customers disproportionately hurt the poor. In terms of environmental impact, consumers are incentivized by marketing appeals to reduce consumption of non-essential products, but then might use the money saved to buy other, unsustainable goods, negating the resource-saving benefit of reduced consumption, due to so-called rebound effects (Yakobovitch & Grinstein, 2016). But this effect is impossible to control by a single business.

For marketing researchers interested in transformative marketing approaches in the age of the Anthropocene, Integrative Social Contracts Theory (Donaldson & Dunfee, 1994) can serve as a framework for addressing ethical issues arising from organisations' global relationships with various stakeholders and their cross-cultural activities. The theory includes two different types of social contracts: a hypothetical macro-social contract and actual micro-social contracts based on living communities (Dunfee et al., 1999). An example of a social contract in a living community designed to promote regeneration could be the creation of local agreements that focus on sustainable practices and community wellbeing. The priority rules for normative judgments derived from the basic assumptions of the macro-social contract can guide marketers and managers in determining what is right and wrong and what they should do when confronted with an ethical problem (Dunfee, 2006). The macro-social contract then sets the boundaries for the micro-social contract which can practically translate into elements such as high levels of service, warrantees, and repair and maintenance options, to drive sufficiency.

Finally, there is an important role for policy-makers to support the dismantling of the Bad Anthropocene. First, based on scientific evidence on environmental footprints, policy-makers could formulate the safe consumption corridors or lower and upper boundaries of consumption, where the latter is of particular importance in the Global Hemisphere (Akenji et al., 2021; Bärnthaler, 2024; Fuchs et al., 2021). This can start as a guidance, but be followed up with taxes or personal carbon trading schemes (Bocken et al., 2022). Second, policy can phase out the most unsustainable products and sectors from the market by first removing their subsidies (e.g., fossil fuels, meat production) and using labeling to signal (un)sustainable product characteristics. Simultaneously, in order to build towards the Good Anthropocene, policy should embrace the restoration, preservation and regeneration of the natural environment, biodiversity, and socio-economic systems. This may be enabled by policy-makers moving subsidies towards sustainable sectors like renewable energy or agriculture supporting plant-based diets. Finally, policy-making should start to focus more holistically on the role of business and marketing and critically look at spaces where companies create addictive patterns (e.g., for processed foods, social media) and start to curtail these, similar to banning addictive tactics in alcohol and cigarette use. The 4Ps, like product, place, price and promotion have already been used effectively to reduce cigarette addictions, e.g., by removing cigarettes out of sight, increasing prices and banishing branding. Such tactics could also be applied to an increasing number of unsustainable product categories (Table 8).

## Conclusion

This paper set out to clarify the role of Marketing in the Anthropocene. As things stand, marketing is a for profit endeavour, the main goal being increased consumption. In so far as sustainability concerns feature in marketing, they are there to fulfil the purpose of profit. However, from the current state of the world, it is clear that business-as-usual and marketing-as-usual are not viable future pathways. We need a vision of marketing in which social and environmental impact is no longer a marketing strategy, but a moral imperative required for every organisation. Marketing should ultimately aim to do more good and less harm, by acting as a function of larger socio-ecological systems within which it already exists. Marketing should be objective, honest and informative rather than predatory. It should serve the consumer and the ecosystem, not purely profit-oriented business interests. Marketing can be more responsible, fulfil a function for society, and communicate the limits of ecosystems to handle business activities. This can, for example, involve communicating to consumers more directly when any further consumption risks harming ecosystems, or when the capacity of an ecosystem is at its limit, instead inviting further consumption to either be limited or staggered into longer intervals.

The era of Marketing in the Anthropocene therefore has a clear moral and ethical dimension. Ethics serves as a normative moral foundation of marketing and a legitimising force by providing a shared set of values and norms that shape marketing practice as also stipulated by the AMA. Essentially, ethics acts as a moral compass that guides decisions and actions. Without clear legislation, it is often up to the individual marketer to navigate and interpret these ethical principles in their daily work. However, several examples from corporations demonstrate that we cannot expect individual ethics to (fully) align with sustainability. To help marketers make moral decisions and act in the interests of society, they need guidance and qualification, and eventually, legislation. The Marketing in the Anthropocene, which we present in this study, can help to provide this initial moral compass.

This research set out to put forward an inspirational and forward-looking concept of Marketing in the Anthropocene. The marketing ‘toolbox’ of theories, tools and instruments is already comprehensive from a more traditional perspective but also a more novel radical perspective. We end with a call for action for future marketers and researchers to seek inspiration in the rich field of marketing to build towards the novel and forward-looking concept of Marketing in the Anthropocene to address the grand environmental and societal challenges of today.

## Data Availability

Not applicable.

## Appendix

Table 9

**Table 9:** Final sample of 63 papers

1	Abu Bakar, M. F., Wu, W., Proverbs, D., & Mavritsaki, E. (2021). Effective communication for water resilient communities: A conceptual framework. *Water*, 13(20), 2880
2	Ardley, B., & May, C. (2020). Ethical marketer and sustainability: Facing the challenges of overconsumption and the market. *Strategic Change*, 29(6), 617–624
3	Armstrong Soule, C. A., & Reich, B. J. (2015). Less is more: is a green demarketing strategy sustainable?. *Journal of Marketing Management*, 31(13–14), 1403–1427
4	Balderjahn, I., & Appenfeller, D. (2023). A social marketing approach to voluntary simplicity: Communicating to consume less. *Sustainability*, 15(3), 2302
5	Balderjahn, I., & Hoffmann, S. (2023). The Effectiveness of Consume-less Appeals in Social Marketing. *Journal of Macromarketing*, 02761467231205448
6	Bocken, N. (2017). Business-led sustainable consumption initiatives: Impacts and lessons learned. *Journal of Management Development*, 36(1), 81–96
7	Bocken, N. M., & Allwood, J. M. (2012). Strategies to reduce the carbon footprint of consumer goods by influencing stakeholders. *Journal of Cleaner Production*, 35, 118–129
8	Chen, W. F., & Liu, J. (2023). When less is more: Understanding consumers' responses to minimalist appeals. *Psychology & Marketing*, 40(10), 2151–2162
9	Connelly, B. L., Ketchen, D. J., & Slater, S. F. (2011). Toward a “theoretical toolbox” for sustainability research in marketing. *Journal of the Academy of Marketing Science*, 39, 86–100
10	Ertekin, Z., & Atik, D. (2015). Sustainable markets: Motivating factors, barriers, and remedies for mobilization of slow fashion. *Journal of Macromarketing*, 35(1), 53–69
11	Frick, V., Gossen, M., Santarius, T., & Geiger, S. (2021). When your shop says# lessismore. Online communication interventions for clothing sufficiency. *Journal of Environmental Psychology*, 75, 101,595
12	Frick, V., Matthies, E., Thøgersen, J., & Santarius, T. (2020). Do online environments promote sufficiency or overconsumption? Online advertisement and social media effects on clothing, digital devices, and air travel consumption. *Journal of Consumer Behaviour*, 20(2), 288–308
13	García-de-Frutos, N., Ortega-Egea, J. M., & Martínez-del-Río, J. (2018). Anti-consumption for environmental sustainability: Conceptualization, review, and multilevel research directions. *Journal of Business Ethics*, 148, 411–435
14	Garcia-Ortega, B., Galan-Cubillo, J., Llorens-Montes, F. J., & de-Miguel-Molina, B. (2023). Sufficient consumption as a missing link toward sustainability: The case of fast fashion. *Journal of Cleaner Production*, 399, 136,678
15	Gorge, H., Herbert, M., Özçağlar-Toulouse, N., & Robert, I. (2015). What do we really need? Questioning consumption through sufficiency. *Journal of Macromarketing*, 35(1), 11–22
16	Gossen, M., & Heinrich, A. (2021). Encouraging consumption reduction: Findings of a qualitative study with clothing companies on sufficiency-promoting communication. *Cleaner and Responsible Consumption*, 3, 100028
17	Gossen, M., & Kropfeld, M. I. (2022). “Choose nature. Buy less.” Exploring sufficiency-oriented marketing and consumption practices in the outdoor industry. *Sustainable Production and Consumption*, 30, 720–736
18	Gossen, M., Ziesemer, F., & Schrader, U. (2019). Why and how commercial marketing should promote sufficient consumption: A systematic literature review. *Journal of Macromarketing*, 39(3), 252–269
19	Haider, M., Shannon, R., & Moschis, G. P. (2022). Sustainable consumption research and the role of marketing: A review of the literature (1976–2021). *Sustainability*, 14(7), 3999
20	Hall, C. M. (2018). Climate change and marketing: Stranded research or a sustainable development?. *Journal of Public Affairs*, 18(4), e1893
21	Helm, S. V., Little, V. J., & Frethey-Bentham, C. (2023). “No Marketing on a Dead Planet”: Rethinking Marketing Education to Support a Restoration Economy. *Journal of Macromarketing*, *44*(2), 307–323
22	Hesse, A., & Rünz, S. (2022). ‘Fly Responsibly’: a case study on consumer perceptions of a green demarketing campaign. *Journal of Marketing Communications*, 28(3), 232–252
23	Hwang, C., Lee, Y., Diddi, S., & Karpova, E. (2016). “Don’t buy this jacket” Consumer reaction toward anti-consumption apparel advertisement. *Journal of Fashion Marketing and Management: An International Journal*, 20(4), 435–452
24	Jutbring, H. (2018). Social marketing through a music festival: Value perceived by festival visitors who reduced meat consumption. *Journal of Social Marketing*, 8(2), 237–256
25	Kamila, M. K., & Jasrotia, S. S. (2023). Ethics and marketing responsibility: A bibliometric analysis and literature review. *Asia Pacific Management Review, **28*(4), 567–583
26	Kelleci, A., & Yıldız, O. (2021). A guiding framework for levels of sustainability in marketing. *Sustainability*, 13(4), 1644
27	Kemper, J. A., & Ballantine, P. W. (2019). What do we mean by sustainability marketing?. *Journal of Marketing Management*, 35(3–4), 277–309
28	Kim, S., Ko, E., & Kim, S. J. (2018). Fashion brand green demarketing: Effects on customer attitudes and behavior intentions. *Journal of Global Fashion Marketing*, 9(4), 364–378
29	Kollmuss, A., & Agyeman, J. (2002). Mind the gap: why do people act environmentally and what are the barriers to pro-environmental behavior?. *Environmental Education Research*, 8(3), 239–260
30	Kotler, P. (2011). Reinventing marketing to manage the environmental imperative. *Journal of Marketing*, 75(4), 132–135
31	Lawrence, J., & Mekoth, N. (2023). Demarketing for sustainability: A review and future research agenda. *International Journal of Consumer Studies*, 47(6), 2157–2180
32	Lim, W. M. (2016). A blueprint for sustainability marketing: Defining its conceptual boundaries for progress. *Marketing Theory*, 16(2), 232–249
33	Lim, W. M. (2017). Inside the sustainable consumption theoretical toolbox: Critical concepts for sustainability, consumption, and marketing. *Journal of Business Research*, 78, 69–80
34	Little, V. J., Lee, C. K. C., & Nair, S. (2019). Macro-demarketing: the key to unlocking unsustainable production and consumption systems?. *Journal of Macromarketing*, 39(2), 166–187
35	Lloveras, J., Marshall, A. P., Vandeventer, J. S., & Pansera, M. (2022). Sustainability marketing beyond sustainable development: towards a degrowth agenda. *Journal of Marketing Management*, 38(17–18), 2055–2077
36	Lowe, B., Lynch, D., & Lowe, J. (2015). Reducing household water consumption: a social marketing approach. *Journal of Marketing Management*, 31(3–4), 378–408
37	Lunde, M. B. (2018). Sustainability in marketing: A systematic review unifying 20 years of theoretical and substantive contributions (1997–2016). *AMS review*, 8(3), 85–110
38	Majer, J. M., Henscher, H. A., Reuber, P., Fischer-Kreer, D., & Fischer, D. (2022). The effects of visual sustainability labels on consumer perception and behavior: A systematic review of the empirical literature. *Sustainable Production and Consumption*, 33, 1–14
39	McDonagh, P., & Prothero, A. (2014). Sustainability marketing research: Past, present and future. *Journal of Marketing Management*, 30(11–12), 1186–1219
40	Milani, P., Carnahan, E., Kapoor, S., Ellison, C., Manus, C., Spohrer, R., van den Berg, G., Wolfson, J. & Kreis, K. (2017). Social marketing of a fortified staple food at scale: generating demand for fortified rice in Brazil. *Journal of Food Products Marketing*, 23(8), 955–978
41	Mohamed Sadom, N. Z., Quoquab, F., Mohammad, J., & Hussin, N. (2020). Less is more: the role of frugality in the Malaysian hotel industry. *International Journal of Tourism Cities*, 8(1), 260–285
42	Morales, P. A., True, S., & Tudor, R. K. (2020). Insights, challenges and recommendations for research on sustainability in marketing. *Journal of Global Scholars of Marketing Science*, 30(4), 394–406
43	Niessen, L., Bocken, N. M., & Dijk, M. (2023). Sufficiency as trend or tradition?—Uncovering business pathways to sufficiency through historical advertisements. *Frontiers in Sustainability*, 4, 1,165,682
44	Park, J. Y., Perumal, S. V., Sanyal, S., Ah Nguyen, B., Ray, S., Krishnan, R., Narasimhaiah, R. & Thangam, D. (2022). Sustainable marketing strategies as an essential tool of business. *American Journal of Economics and Sociology*, 81(2), 359–379
45	Peattie, K. (2001). Towards sustainability: The third age of green marketing. *The Marketing Review*, 2(2), 129–146
46	Peattie, K., & Peattie, S. (2009). Social marketing: a pathway to consumption reduction?. *Journal of Business Research*, 62(2), 260–268
47	Peattie, K., Peattie, S., & Ponting, C. (2009). Climate change: a social and commercial marketing communications challenge. *EuroMed Journal of Business*, 4(3), 270–286
48	Pereira Heath, M. T., & Chatzidakis, A. (2012). ‘Blame it on marketing’: consumers' views on unsustainable consumption. *International Journal of Consumer Studies*, 36(6), 656–667
49	Pham, H., Dang, H. P., & Nguyen-Viet, B. (2023). How can CSR in demarketing trigger brand advocacy and mindful consumption? Mediating roles of perceived corporate hypocrisy and brand credibility. *Journal of Fashion Marketing and Management: An International Journal*, 27(5), 851–869
50	Pinto, O., & Casais, B. (2023). Multilevel implications for anti-consumption social marketing within the public policy framework for SDG realization: a systematic literature review. *International Review on Public and Nonprofit Marketing*, 20(3), 605–634
51	Prothero, A., & McDonagh, P. (2021). Ambiguity of purpose and the politics of failure: Sustainability as macromarketing’s compelling political calling. *Journal of Macromarketing*, 41(1), 166–171
52	Raab, K., Wagner, R., Ertz, M., & Salem, M. (2023). When marketing discourages consumption: demarketing of single-use plastics for city tourism in Ottawa, Canada. *Journal of Ecotourism*, 22(3), 375–405
53	Ramirez, E., Tajdini, S., & David, M. E. (2017). The effects of proenvironmental demarketing on consumer attitudes and actual consumption. *Journal of Marketing Theory and Practice*, 25(3), 291–304
54	Rashidi-Sabet, S., & Madhavaram, S. (2022). A strategic marketing framework for emerging out of the climate change social trap: the case of the fashion industry. *Journal of Macromarketing*, 42(2), 267–291
55	Reich, B. J., & Soule, C. A. A. (2016). Green demarketing in advertisements: Comparing “buy green” and “buy less” appeals in product and institutional advertising contexts. *Journal of Advertising*, 45(4), 441–458
56	Seegebarth, B., Peyer, M., Balderjahn, I., & Wiedmann, K. P. (2016). The sustainability roots of anticonsumption lifestyles and initial insights regarding their effects on consumers' well‐being. *Journal of Consumer Affairs*, 50(1), 68–99
57	Sekhon, T. S., & Armstrong Soule, C. A. (2020). Conspicuous anticonsumption: When green demarketing brands restore symbolic benefits to anticonsumers. *Psychology & Marketing*, 37(2), 278–290
58	Sharma, Y., & Silal, P. (2023). Mapping the effect of healthy and unhealthy food and beverages marketing: two decades of bibliometric analysis. *European Journal of Marketing*, 57(1), 149–184
59	Sodhi, K. (2011). Has marketing come full circle? Demarketing for sustainability. *Business Strategy Series*, 12(4), 177–185
60	Solér, C. (2012). Conceptualizing sustainably produced food for promotional purposes: A sustainable marketing approach. *Sustainability*, 4(3), 294–340
61	Varey, R. J. (2010). Marketing means and ends for a sustainable society: A welfare agenda for transformative change. *Journal of Macromarketing*, 30(2), 112–126
62	Varey, R. J. (2012). The marketing future beyond the limits of growth. *Journal of Macromarketing*, 32(4), 424–433
63	Yakobovitch, N., & Grinstein, A. (2016). Materialism and the boomerang effect of descriptive norm demarketing: Extension and remedy in an environmental context. *Journal of Public Policy & Marketing*, 35(1), 91–107

## Footnotes

1 While the term “Anthropocene” is not officially recognised by the Union of Geological Scientists, it is considered an important and useful descriptor for the large-scale impact that humanity has on the planet (Carrington, 2024).

2 Available under https://journals.scholarsportal.info/browse/00920703/v39i0001
